# Hybrid Genome Assemblies of 245 Avian and Broiler Barn Environment-Associated Escherichia coli Strains Isolated from Saskatchewan Broiler Farms

**DOI:** 10.1128/mra.00110-23

**Published:** 2023-04-26

**Authors:** Haley Sanderson, Chinenye R. Nnajide, Madeline C. McCarthy, Reema Singh, Joseph E. Rubin, Jo-Anne R. Dillon, Aaron P. White

**Affiliations:** a Vaccine and Infectious Disease Organization (VIDO), University of Saskatchewan, Saskatoon, Saskatchewan, Canada; b Department of Biochemistry, Microbiology and Immunology, University of Saskatchewan, Saskatoon, Saskatchewan, Canada; c Department of Veterinary Microbiology, University of Saskatchewan, Saskatoon, Saskatchewan, Canada; University of Maryland School of Medicine

## Abstract

Escherichia coli infections in poultry cause significant morbidity and economic losses for producers each year. In a 3-year period, we collected and sequenced the whole genomes of E. coli disease isolates (*n* = 91), isolates from presumed healthy birds (*n* = 61), and isolates from 8 barn sites (*n* = 93) on broiler farms in Saskatchewan.

## ANNOUNCEMENT

Avian pathogenic Escherichia coli (APEC) strains cause colibacillosis, a common disease affecting the poultry industry worldwide (1, 2). These infections can be localized or systemic and are associated with gross lesions on the bird’s abdominal and thoracic viscera (3–5). It is important to identify E. coli strains predisposed to increased pathogenicity, especially with the potential for zoonotic transmission to humans (6). We sequenced E. coli isolates from diseased birds, healthy chickens in the same flocks, and 8 different sites on Saskatchewan broiler barns (Table 1).

**TABLE 1:** De novo genome assembly data for 245 avian-associated *E. coli* strains

Strain	Phylogroup^*a*^	GenBank name^*b*^		Illumina sequencing results				ONT sequencing results				Genome size (bp)	No. of contigs	*N*_50_ (bp)	GC content (%)	Assembler	Farm location^*c*^
			GenBank assembly accession no.	No. of reads	Avg read length (bp)	Coverage depth (×)	SRA accession no.	No. of reads	Avg read length (bp)	Coverage depth (×)	SRA accession no.						
0012_1H1	G	EC_0012_1H1_D	GCA_027275285.1	26,349,850	150	758	SRR22784418	104,325	6,629	133	SRR22784417	5,215,630	3	5,079,903	50.6	Unicycler	Rose Valley
0012_2H1	G	EC_0012_2H1_D	GCA_027275955.1	43,254,644	150	1,243	SRR22784727	36,452	7,849	55	SRR22784908	5,219,271	19	4,348,170	50.6	SPAdes	Rose Valley
0012_2L1	B2	EC_0012_2L1_D	GCA_027278825.1	28,582,816	150	849	SRR22784768	190,258	6,035	227	SRR22784767	5,049,921	2	4,903,617	50.6	Unicycler	Rose Valley
0012_3S1	G	EC_0012_3S1_D	GCA_027279255.1	29,900,648	150	858	SRR22784491	112,332	5,856	126	SRR22784490	5,221,291	9	5,080,166	50.6	Unicycler	Rose Valley
0012_C1	A	EC_0012_C1_H	GCA_027274605.1	28,995,274	150	841	SRR22784446	190,388	6,096	224	SRR22784445	5,165,428	5	5,061,833	50.5	Unicycler	Rose Valley
0012_C3	B1	EC_0012_C3_H	GCA_027275335.1	28,909,470	150	846	SRR22784521	903	5,015	1	SRR22784520	5,126,454	163	109,119	50.4	Unicycler	Rose Valley
0012_C5	A	EC_0012_C5_H	GCA_027276285.1	26,731,498	150	776	SRR22784901	164,021	6,751	214	SRR22784900	5,164,099	5	5,060,504	50.5	Unicycler	Rose Valley
0012_C7	B1	EC_0012_C7_H	GCA_027271555.1	26,972,498	150	816	SRR22784500	216,673	5,753	251	SRR22784499	4,952,861	7	4,823,106	50.7	Unicycler	Rose Valley
0038_1H1	Cryptic	EC_0038_1H1_D	GCA_027275735.1	33,335,492	150	1,015	SRR22784635	148,935	6,344	192	SRR22784634	4,927,600	2	4,798,726	50.8	Flye_Medaka_Pilon	Grandora
0038_1S2	B2	EC_0038_1S2_D	GCA_027274885.1	13,076,542	150	373	SRR22784558	49,519	6,889	65	SRR22784557	5,260,889	3	5,121,182	50.6	Unicycler	Grandora
0038_2L3	B2	EC_0038_2L3_D	GCA_027276665.1	43,830,802	150	1,250	SRR22784612	36,150	7,437	51	SRR22784611	5,261,294	3	5,121,630	50.6	Unicycler	Grandora
0038_3S2	B2	EC_0038_3S2_D	GCA_027275225.1	24,919,336	150	710	SRR22784687	42,988	7,965	65	SRR22784686	5,261,381	3	5,121,674	50.6	Unicycler	Grandora
0038_C1	A	EC_0038_C1_H	GCA_027271315.1	9,759,588	150	269	SRR22784785	35,431	6,999	46	SRR22784784	5,434,608	17	5,193,826	50.4	Unicycler	Grandora
0038_C3	A	EC_0038_C3_H	GCA_027273665.1	47,282,862	150	1,306	SRR22784436	16,003	6,188	18	SRR22784435	5,430,615	16	5,188,016	50.5	Unicycler	Grandora
0038_C7	A	EC_0038_C7_H	GCA_027274685.1	68,431,112	150	1,886	SRR22784451	51,594	6,610	63	SRR22784450	5,441,922	41	5,198,982	50.4	SPAdes	Grandora
0038_C9	A	EC_0038_C9_H	GCA_027279005.1	19,360,508	150	527	SRR22784779	46,331	6,153	52	SRR22784778	5,510,013	17	5,193,197	50.4	Unicycler	Grandora
0038_C10	A	EC_0038_C10_H	GCA_027273685.1	45,399,992	150	1,259	SRR22784429	54,889	6,768	69	SRR22784428	5,410,117	19	5,193,832	50.4	Unicycler	Grandora
0038_C11	A	EC_0038_C11_H	GCA_027279545.1	33,545,748	150	926	SRR22784743	115,651	6,357	135	SRR22784732	5,433,804	17	5,195,958	50.4	Unicycler	Grandora
0038_C12	A	EC_0038_C12_H	GCA_027279105.1	30,268,076	150	838	SRR22784802	32,259	6,400	38	SRR22784791	5,415,803	37	4,992,869	50.4	SPAdes	Grandora
0205_1L1	G	EC_0205_1L1_D	GCA_027279065.1	14,942,550	150	428	SRR22784476	56,000	6,226	67	SRR22784475	5,234,812	23	3,500,866	50.7	SPAdes	Hepburn
0205_1L3	G	EC_0205_1L3_D	GCA_027274985.1	33,140,750	150	946	SRR22784565	82,425	3,846	60	SRR22784564	5,249,083	10	5,071,946	50.7	Unicycler	Hepburn
0205_2L2	G	EC_0205_2L2_D	GCA_027273885.1	28,701,332	150	821	SRR22784582	218,158	3,491	145	SRR22784581	5,238,530	9	5,070,155	50.7	Unicycler	Hepburn
0205_2S2	G	EC_0205_2S2_D	GCA_027274765.1	18,630,682	150	530	SRR22784464	20,000	6,784	26	SRR22784463	5,268,495	40	4,614,462	50.7	SPAdes	Hepburn
0205_3H1	D	EC_0205_3H1_D	GCA_027276365.1	29,119,702	150	815	SRR22784734	117,102	9,943	217	SRR22784733	5,351,802	5	5,062,518	50.6	Unicycler	Hepburn
0205_3S1	D	EC_0205_3S1_D	GCA_027275545.1	34,166,828	150	957	SRR22784620	125,264	3,441	80	SRR22784619	5,351,793	5	5,062,509	50.6	Unicycler	Hepburn
0205_C3	A	EC_0205_C3_H	GCA_027271465.1	36,085,116	150	1,023	SRR22784800	228,991	3,792	164	SRR22784799	5,285,434	6	3,734,196	50.4	Unicycler	Hepburn
0205_C4	A	EC_0205_C4_H	GCA_027278085.1	18,828,470	150	534	SRR22784731	134,604	5,747	146	SRR22784730	5,285,353	5	5,111,217	50.4	Unicycler	Hepburn
0205_C6	B2	EC_0205_C6_H	GCA_027278505.1	10,349,318	150	318	SRR22784934	64,564	6,294	83	SRR22784933	4,877,545	2	4,686,401	50.8	Flye_Medaka_Pilon	Hepburn
0205_C7	G	EC_0205_C7_H	GCA_027274475.1	25,557,664	150	619	SRR22784584	183,370	3,786	112	SRR22784583	6,186,773	3	5,024,044	51.0	Unicycler	Hepburn
0205_C9	A	EC_0205_C9_H	GCA_027278365.1	23,862,512	150	680	SRR22784912	189,021	3,669	132	SRR22784911	5,261,702	6	5,070,291	50.5	Unicycler	Hepburn
0731_1H1	G	EC_0731_1H1_D	GCA_027275325.1	20,941,246	150	579	SRR22784523	62,380	11,792	136	SRR22784522	5,426,764	9	5,039,911	50.6	Unicycler	Hepburn
0731_2H1	G	EC_0731_2H1_D	GCA_027278425.1	26,588,808	150	734	SRR22784958	24,070	7,778	34	SRR22784957	5,426,104	27	3,276,808	50.6	SPAdes	Biggar
0731_2S2	G	EC_0731_2S2_D	GCA_027274115.1	32,088,648	150	892	SRR22784420	76,326	13,842	196	SRR22784419	5,395,623	26	1,352,267	50.6	SPAdes	Biggar
0731_3L2	G	EC_0731_3L2_D	GCA_027278585.1	37,575,130	150	1,037	SRR22784478	35,312	12,691	82	SRR22784477	5,437,462	8	5,040,272	50.6	Unicycler	Biggar
0731_SB2C1	B1	EC_0731_SB2C1_H	GCA_027275665.1	27,536,986	150	824	SRR22784623	3,527	6,069	4	SRR22784622	5,012,169	34	396,182	50.6	Unicycler	Biggar
0731_SB3C1	B1	EC_0731_SB3C1_H	GCA_027275865.1	43,912,872	150	1,257	SRR22784872	4,465	5,614	5	SRR22784871	5,239,338	35	436,757	50.6	Unicycler	Biggar
0731_SB3C2	B2	EC_0731_SB3C2_H	GCA_027273985.1	25,829,654	150	728	SRR22784598	2,630	6,343	3	SRR22784597	5,323,671	93	214,455	50.7	Unicycler	Biggar
23315_H1	G	EC_23315_H1_D	GCA_027278985.1	21,529,536	150	605	SRR22784527	14,094	8,506	22	SRR22784516	5,332,439	34	1,517,281	50.6	SPAdes	Grandora-2
23315_L3	G	EC_23315_L3_D	GCA_027274125.1	31,950,198	150	894	SRR22784587	17,292	7,354	24	SRR22784586	5,358,825	3	5,126,324	50.6	Unicycler	Grandora-2
23315_S2	G	EC_23315_S2_D	GCA_027273735.1	33,377,358	150	925	SRR22784794	127,844	4,241	100	SRR22784793	5,406,872	12	5,126,333	50.6	Unicycler	Grandora-2
23315_C1	A	EC_23315_C1_H	GCA_027275625.1	26,437,620	150	720	SRR22784627	64,405	7,213	84	SRR22784626	5,505,482	28	3,771,415	50.4	Unicycler	Grandora-2
23315_C2	A	EC_23315_C2_H	GCA_027276025.1	44,476,892	150	1,239	SRR22784649	24,951	7,139	33	SRR22784648	5,386,626	73	741,867	50.4	Unicycler	Grandora-2
23315_C3	A	EC_23315_C3_H	GCA_027275295.1	36,867,364	150	996	SRR22784532	58,385	8,304	87	SRR22784531	5,553,881	9	5,337,680	50.4	Unicycler	Grandora-2
23315_C4	C	EC_23315_C4_H	GCA_027275485.1	40,297,732	150	1,171	SRR22784745	138,272	11,592	310	SRR22784744	5,474,589	7	4,813,788	50.7	Unicycler	Grandora-2
23315_C5	C	EC_23315_C5_H	GCA_027275265.1	25,097,806	150	730	SRR22784416	7,599	8,253	12	SRR22784415	5,153,013	9	4,288,702	50.7	Unicycler	Grandora-2
23315_C8	A	EC_23315_C8_H	GCA_027275445.1	30,593,430	150	829	SRR22784423	69,414	12,142	152	SRR22784422	5,538,493	3	5,342,020	50.4	Flye_Medaka_Pilon	Grandora-2
23315_C9	A	EC_23315_C9_H	GCA_027278545.1	33,969,336	150	929	SRR22784936	86,932	5,901	94	SRR22784935	5,479,002	25	3,270,672	50.5	Unicycler	Grandora-2
2402_2L1	G	EC_2402_2L1_D	GCA_027274025.1	34,514,840	150	987	SRR22784442	177,888	3,846	130	SRR22784441	5,237,806	7	3,160,862	50.8	Unicycler	Herbert
2402_3H2	G	EC_2402_3H2_D	GCA_027279425.1	45,441,292	150	1,288	SRR22784726	24,862	7,426	35	SRR22784715	5,289,548	22	5,044,096	50.7	SPAdes	Herbert
2402_3S1	G	EC_2402_3S1_D	GCA_027278845.1	27,759,544	150	777	SRR22784761	81,719	4,007	61	SRR22784760	5,352,732	7	5,060,789	50.8	Unicycler	Herbert
2402_C1	A	EC_2402_C1_H	GCA_027273715.1	16,350,100	150	477	SRR22784434	86,866	7,824	132	SRR22784433	5,139,064	22	4,730,383	50.4	Unicycler	Herbert
2655_1L3	G	EC_2655_1L3_D	GCA_027274745.1	43,434,812	150	1,203	SRR22784543	95,972	11,496	204	SRR22784542	5,416,051	25	1,860,373	50.8	SPAdes	Birch Hills
2655_1S2	A	EC_2655_1S2_D	GCA_027278325.1	24,284,752	150	739	SRR22784919	98,168	11,776	234	SRR22784918	4,929,665	14	3,641,999	50.8	SPAdes	Birch Hills
2655_2H3	A	EC_2655_2H3_D	GCA_027271585.1	10,430,948	150	306	SRR22784787	29,268	7,961	46	SRR22784786	5,120,194	18	4,696,749	50.8	Unicycler	Birch Hills
2655_2L2	A	EC_2655_2L2_D	GCA_027279315.1	42,828,552	150	1,301	SRR22784539	25,469	13,427	69	SRR22784432	4,934,868	21	4,581,672	50.8	SPAdes	Birch Hills
2655_2S2	A	EC_2655_2S2_D	GCA_027271665.1	15,705,586	150	478	SRR22784811	26,578	11,584	62	SRR22784810	4,932,247	12	4,702,991	50.8	SPAdes	Birch Hills
3862_S1	G	EC_3862_L1_D	GCA_027279305.1	32,815,100	150	937	SRR22784959	79,979	12,770	194	SRR22784948	5,250,128	4	5,053,148	50.8	Unicycler	Dalmeny
3862_L1	G	EC_3862_S1_D	GCA_027274835.1	23,132,904	150	661	SRR22784549	173,641	11,879	393	SRR22784548	5,247,221	3	5,052,620	50.8	Unicycler	Dalmeny
3862_H1	Cryptic	EC_3862_H1_D	GCA_027276235.1	36,357,808	150	1,142	SRR22784884	127,054	10,498	279	SRR22784883	4,776,983	3	4,559,620	50.9	Flye_Medaka_Pilon	Dalmeny
3862_S2	G	EC_3862_S2_D	GCA_027275995.1	38,148,082	150	1,120	SRR22784645	1,216	8,451	2	SRR22784643	5,103,893	136	197,395	50.7	Unicycler	Dalmeny
3862_C2	E	EC_3862_C2_H	GCA_027276145.1	29,507,590	150	753	SRR22784890	69,972	12,021	143	SRR22784889	5,877,860	4	5,655,776	50.1	Unicycler	Dalmeny
3862_C8	A	EC_3862_C8_H	GCA_027278525.1	34,129,676	150	997	SRR22784945	109,758	6,097	130	SRR22784944	5,135,786	3	5,040,202	50.5	Unicycler	Dalmeny
3862_C11	E	EC_3862_C11_H	GCA_027274905.1	37,080,158	150	948	SRR22784822	32,000	8,213	45	SRR22784821	5,866,206	7	3,442,299	50.1	Unicycler	Dalmeny
4957_1H1	A	EC_4957_1H1_D	GCA_027271305.1	35,330,268	150	972	SRR22784789	84,000	8,482	131	SRR22784788	5,444,550	8	5,105,559	50.8	Unicycler	Osler
4957_1H2	B2	EC_4957_1H2_D	GCA_027276805.1	19,607,174	150	545	SRR22784723	121,417	5,040	113	SRR22784722	5,390,410	13	3,671,766	50.5	Unicycler	Osler
4957_2L1	A	EC_4957_2L1_D	GCA_027276385.1	35,993,816	150	1,003	SRR22784569	8,538	7,863	12	SRR22784568	5,384,642	11	1,819,896	50.7	Flye_Medaka_Pilon	Osler
4957_2L3	B2	EC_4957_2L3_D	GCA_027274065.1	25,927,040	150	704	SRR22784455	64,000	8,540	99	SRR22784454	5,521,365	6	5,120,130	50.6	Unicycler	Osler
4957_3S1	A	EC_4957_3S1_D	GCA_027273855.1	38,765,100	150	1,020	SRR22784831	172,000	8,500	256	SRR22784828	5,700,474	4	5,137,439	50.5	Flye_Medaka_Pilon	Osler
4957_C1	A	EC_4957_C1_H	GCA_027278175.1	47,273,526	150	1,401	SRR22784738	128,000	8,193	207	SRR22784737	5,055,008	2	4,933,024	50.7	Unicycler	Osler
4957_C3	G	EC_4957_C3_H	GCA_027278235.1	38,883,690	150	1,100	SRR22784924	88,000	7,889	131	SRR22784923	5,296,508	4	5,058,920	50.8	Unicycler	Osler
4957_C6	A	EC_4957_C6_H	GCA_027274365.1	23,362,630	150	670	SRR22784805	163,333	13,029	407	SRR22784804	5,227,681	2	5,110,197	50.6	Unicycler	Osler
4984_6B1	G	EC_4984_6B1_D	GCA_027278725.1	27,552,022	150	767	SRR22784765	42,738	8,381	66	SRR22784764	5,387,085	26	1,190,529	50.5	SPAdes	Saskatoon
4984_6H1	G	EC_4984_6H1_D	GCA_027276155.1	33,014,014	150	919	SRR22784898	56,075	7,617	79	SRR22784897	5,389,805	8	5,245,044	50.5	Unicycler	Saskatoon
6041_1L2	D	EC_6041_1L2_D	GCA_027278925.1	44,648,652	150	1,233	SRR22784485	188,750	5,686	198	SRR22784484	5,431,221	4	5,027,479	50.6	Unicycler	Waldheim
6041_2L1	D	EC_6041_2L1_D	GCA_027274995.1	12,389,906	150	342	SRR22784813	124,000	6,475	148	SRR22784566	5,432,146	10	5,029,323	50.6	Unicycler	Waldheim
6041_3L1	G	EC_6041_3L1_D	GCA_027276825.1	12,341,308	150	354	SRR22784614	32,000	7,022	43	SRR22784613	5,233,728	6	4,931,750	50.7	Unicycler	Waldheim
6041_3S1	G	EC_6041_3S1_D	GCA_027276565.1	29,112,370	150	834	SRR22784468	92,000	6,326	111	SRR22784467	5,234,315	6	4,932,294	50.7	Unicycler	Waldheim
6041_C1	A	EC_6041_C1_H	GCA_027276705.1	16,993,734	150	485	SRR22784845	82,893	6,580	104	SRR22784630	5,260,539	27	3,852,184	50.4	SPAdes	Waldheim
6041_C2	A	EC_6041_C2_H	GCA_027276165.1	34,682,898	150	1,032	SRR22784879	60,000	6,881	82	SRR22784878	5,040,603	5	4,876,116	50.5	Flye_Medaka_Pilon	Waldheim
6041_C5	A	EC_6041_C5_H	GCA_027274445.1	24,789,526	150	728	SRR22784528	60,312	7,173	85	SRR22784526	5,109,495	4	5,003,632	50.4	Unicycler	Waldheim
6041_C6	C	EC_6041_C6_H	GCA_027275425.1	29,185,818	150	867	SRR22784408	88,000	6,366	111	SRR22784407	5,051,190	3	4,950,011	50.5	Flye_Medaka_Pilon	Waldheim
6041_C7	A	EC_6041_C7_H	GCA_027279045.1	12,733,950	150	365	SRR22784504	63,397	6,933	84	SRR22784685	5,232,963	14	3,319,424	50.4	SPAdes	Waldheim
6041_C9	A	EC_6041_C9_H	GCA_027279145.1	33,578,398	150	962	SRR22784489	84,000	5,937	95	SRR22784488	5,234,267	4	5,025,869	50.5	Unicycler	Waldheim
6041_C10	A	EC_6041_C10_H	GCA_027279205.1	20,172,832	150	576	SRR22784633	31,450	8,365	50	SRR22784868	5,255,369	18	3,291,030	50.4	SPAdes	Waldheim
6245_1H1	C	EC_6245_1H1_D	GCA_027271385.1	28,571,318	150	856	SRR22784665	188,552	6,949	262	SRR22784664	5,001,931	4	4,782,495	50.7	Unicycler	Shaunavon
6245_1L1	A	EC_6245_1L1_D	GCA_027275365.1	28,507,760	150	859	SRR22784534	105,398	7,005	148	SRR22784533	4,975,419	3	4,759,324	50.6	Flye_Medaka_Pilon	Shaunavon
6245_1S2	C	EC_6245_1S2_D	GCA_027279125.1	40,080,258	150	1,200	SRR22784494	133,551	6,757	180	SRR22784493	5,002,684	4	4,782,343	50.6	Unicycler	Shaunavon
6245_2L1	C	EC_6245_2L1_D	GCA_027279365.1	32,108,678	150	962	SRR22784880	236,285	6,369	301	SRR22784655	5,001,918	4	4,782,482	50.7	Unicycler	Shaunavon
6245_3H2	G	EC_6245_3H2_D	GCA_027274505.1	31,407,326	150	869	SRR22784591	33,878	7,954	50	SRR22784590	5,415,497	11	5,046,977	50.5	Unicycler	Shaunavon
6245_3L1	G	EC_6245_3L1_D	GCA_027279475.1	28,307,878	150	783	SRR22784704	210,784	6,842	266	SRR22784469	5,415,500	11	5,046,980	50.5	Unicycler	Shaunavon
6245_C1	C	EC_6245_C1_H	GCA_027274725.1	24,301,622	150	743	SRR22784697	46,582	7,293	69	SRR22784696	4,904,445	3	2,733,891	50.7	Flye_Medaka_Pilon	Shaunavon
6245_C4	B1	EC_6245_C4_H	GCA_027276685.1	26,337,878	150	782	SRR22784617	138,760	7,321	201	SRR22784616	5,045,323	3	4,746,154	50.7	Unicycler	Shaunavon
6245_C5	A	EC_6245_C5_H	GCA_027275385.1	39,950,186	150	1,209	SRR22784536	64,579	8,513	111	SRR22784535	4,951,618	2	4,852,374	50.6	Unicycler	Shaunavon
6245_C6	D	EC_6245_C6_H	GCA_027274645.1	35,018,502	150	869	SRR22784703	59,593	8,330	82	SRR22784702	6,044,658	6	5,806,711	50.6	Flye_Medaka_Pilon	Shaunavon
7360_1S1	G	EC_7360_1S1_D	GCA_027273565.1	37,151,652	150	1,038	SRR22784554	26,073	7,279	35	SRR22784553	5,365,007	31	1,981,473	50.5	SPAdes	Biggar-2
7360_2H2	B1	EC_7360_2H2_D	GCA_027276545.1	36,207,362	150	934	SRR22784717	26,924	6,371	29	SRR22784716	5,817,001	62	638,551	50.6	Unicycler	Biggar-2
7360_3H1	G	EC_7360_3H1_D	GCA_027275725.1	24,526,486	150	690	SRR22784854	55,689	7,012	73	SRR22784853	5,328,789	27	988,512	50.5	SPAdes	Biggar-2
7360_C1	B1	EC_7360_C1_H	GCA_027275005.1	33,867,450	150	993	SRR22784563	1,806	6,321	2	SRR22784562	5,115,502	141	85,644	50.6	Unicycler	Biggar-2
7360_C2	D	EC_7360_C2_H	GCA_027274105.1	28,872,006	150	861	SRR22784673	996	5,834	1	SRR22784672	5,027,442	88	158,039	50.5	Unicycler	Biggar-2
7360_C3	A	EC_7360_C3_H	GCA_027278185.1	24,840,482	150	707	SRR22784941	1,201	6,409	1	SRR22784940	5,267,610	153	126,965	50.4	Unicycler	Biggar-2
7578_1H1	B1	EC_7578_1H1_D	GCA_027276265.1	38,340,458	150	1,147	SRR22784888	103,290	5,855	121	SRR22784887	5,012,343	5	2,375,549	50.4	Unicycler	Vanscoy
7578_1L2	B1	EC_7578_1L2_D	GCA_027278685.1	32,580,552	150	972	SRR22784962	84,259	11,648	195	SRR22784961	5,022,471	2	4,907,800	50.4	Unicycler	Vanscoy
7578_2H2	A	EC_7578_2H2_D	GCA_027273805.1	32,189,206	150	884	SRR22784815	20,000	7,014	26	SRR22784814	5,456,213	21	1,302,211	50.6	Unicycler	Vanscoy
7578_2H3	D	EC_7578_2H3_D	GCA_027273605.1	29,093,604	150	787	SRR22784427	92,369	11,455	191	SRR22784426	5,537,137	14	3,951,325	50.6	Unicycler	Vanscoy
7578_2L2	A	EC_7578_2L2_D	GCA_027279485.1	35,047,446	150	962	SRR22784621	115,657	5,897	125	SRR22784610	5,462,791	6	2,733,818	50.7	Flye_Medaka_Pilon	Vanscoy
7578_3S1	B1	EC_7578_3S1_D	GCA_027275095.1	32,444,628	150	986	SRR22784706	52,509	7,048	75	SRR22784705	4,934,349	2	4,819,684	50.4	Flye_Medaka_Pilon	Vanscoy
8630_1H1	B2	EC_8630_1H1_D	GCA_027279285.1	58,209,614	150	1,619	SRR22784937	34,088	8,624	54	SRR22784926	5,394,712	5	5,190,805	50.5	Unicycler	Grandora-2
8630_1L1	B2	EC_8630_1L1_D	GCA_027278225.1	45,750,816	150	1,228	SRR22784691	29,450	7,718	41	SRR22784690	5,584,318	59	4,357,443	50.5	SPAdes	Grandora-2
8630_1S1	B2	EC_8630_1S1_D	GCA_027273615.1	51,072,876	150	1,420	SRR22784431	18,266	6,750	23	SRR22784430	5,394,684	5	5,190,777	50.5	Unicycler	Grandora-2
8630_1S2	B2	EC_8630_1S2_D	GCA_027274925.1	46,174,572	150	1,245	SRR22784820	36,344	6,489	42	SRR22784819	5,564,865	20	5,196,725	50.5	Unicycler	Grandora-2
8630_3H2	G	EC_8630_3H2_D	GCA_027275235.1	25,646,226	150	709	SRR22784712	53,358	7,368	72	SRR22784711	5,427,007	2	5,309,339	50.7	Unicycler	Grandora-2
8630_C1	E	EC_8630_C1_H	GCA_027275745.1	33,439,974	150	925	SRR22784856	903	6,321	1	SRR22784855	5,424,111	342	78,493	50.2	Unicycler	Grandora-2
8630_C3	D	EC_8630_C3_H	GCA_027274375.1	23,621,396	150	685	SRR22784682	821	5,812	1	SRR22784681	5,169,902	93	144,314	50.4	Unicycler	Grandora-2
9226_1H3	B2	EC_9226_1H3_D	GCA_027276575.1	20,646,360	150	614	SRR22784609	67,976	8,524	115	SRR22784608	5,045,924	4	4,906,109	50.6	Unicycler	Wakaw
9226_1L2	D	EC_9226_1L2_D	GCA_027279025.1	32,810,256	150	853	SRR22784474	72,080	6,374	80	SRR22784473	5,762,607	7	5,455,034	50.5	Unicycler	Wakaw
9226_1S1	A	EC_9226_1S1_D	GCA_027275455.1	33,022,798	150	947	SRR22784792	62,294	6,037	72	SRR22784790	5,223,927	6	4,141,800	50.7	Unicycler	Wakaw
9226_2H2	D	EC_9226_2H2_D	GCA_027273705.1	45,448,176	150	1,293	SRR22784541	52,000	7,705	76	SRR22784540	5,268,945	11	4,954,237	50.6	Unicycler	Wakaw
9226_2L1	D	EC_9226_2L1_D	GCA_027278385.1	30,445,654	150	848	SRR22784927	102,016	6,334	120	SRR22784925	5,382,802	15	4,954,224	50.7	Unicycler	Wakaw
9226_2S1	D	EC_9226_2S1_D	GCA_027276075.1	37,015,586	150	1,024	SRR22784654	40,841	5,015	38	SRR22784653	5,418,323	16	3,786,225	50.6	Unicycler	Wakaw
9226_3H1	D	EC_9226_3H1_D	GCA_027274185.1	41,017,678	150	1,179	SRR22784512	52,800	5,636	57	SRR22784511	5,215,192	9	4,953,954	50.6	Unicycler	Wakaw
9226_3L1	D	EC_9226_3L1_D	GCA_027279445.1	30,706,104	150	881	SRR22784915	61,225	5,689	67	SRR22784754	5,224,330	8	4,954,245	50.7	Unicycler	Wakaw
9226_3S1	D	EC_9226_3S1_D	GCA_027273925.1	24,963,698	150	719	SRR22784576	64,687	8,830	110	SRR22784575	5,209,005	6	4,954,388	50.7	Unicycler	Wakaw
9226_C1	D	EC_9226_C1_H	GCA_027278165.1	21,003,638	150	585	SRR22784755	86,245	3,673	59	SRR22784753	5,377,609	18	4,964,124	50.7	Unicycler	Wakaw
9226_C2	D	EC_9226_C2_H	GCA_027275905.1	36,478,674	150	1,038	SRR22784659	187,076	7,032	250	SRR22784658	5,265,993	4	4,964,460	50.7	Unicycler	Wakaw
9226_C5	B1	EC_9226_C5_H	GCA_027278465.1	36,955,322	150	1,027	SRR22784932	95,557	7,615	135	SRR22784931	5,390,632	6	5,122,182	50.7	Unicycler	Wakaw
9413_1H1	A	EC_9413_1H1_D	GCA_027278055.1	17,170,600	150	453	SRR22784729	130,056	4,949	113	SRR22784728	5,690,224	24	2,293,090	50.7	Unicycler	Saskatoon-2
9413_1S2	A	EC_9413_1S2_D	GCA_027278785.1	19,765,382	150	561	SRR22784956	68,000	6,417	83	SRR22784955	5,284,759	64	208,665	50.5	Unicycler	Saskatoon-2
9413_2H1	A	EC_9413_2H1_D	GCA_027275505.1	27,380,108	150	790	SRR22784680	132,000	6,722	171	SRR22784679	5,195,997	3	4,861,258	50.5	Unicycler	Saskatoon-2
9413_2S2	A	EC_9413_2S2_D	GCA_027276875.1	19,611,308	150	566	SRR22784850	60,000	7,700	89	SRR22784849	5,199,922	4	4,861,182	50.5	Unicycler	Saskatoon-2
9413_3L1	B2	EC_9413_3L1_D	GCA_027278945.1	16,082,222	150	447	SRR22784472	40,012	6,185	46	SRR22784471	5,392,535	9	3,389,450	50.5	Unicycler	Saskatoon-2
9413_3S1	B2	EC_9413_3S1_D	GCA_027279085.1	26,212,518	150	729	SRR22784487	150,227	10,127	282	SRR22784486	5,386,559	7	5,028,159	50.5	Unicycler	Saskatoon-2
9413_C1	G	EC_9413_C1_H	GCA_027276725.1	26,482,772	150	779	SRR22784863	112,000	7,713	169	SRR22784862	5,094,799	112	244,409	50.7	Unicycler	Saskatoon-2
9413_C3	B1	EC_9413_C3_H	GCA_027276525.1	15,818,368	150	483	SRR22784695	59,785	4,866	59	SRR22784470	4,910,611	7	1,348,777	50.7	SPAdes	Saskatoon-2
9413_C4	B1	EC_9413_C4_H	GCA_027276425.1	34,582,912	150	1,013	SRR22784567	80,155	11,117	174	SRR22784844	5,113,471	7	5,018,673	50.7	Unicycler	Saskatoon-2
9503_1H3	B2	EC_9503_1H3_D	GCA_027274275.1	37,868,266	150	1,090	SRR22784530	87,937	6,235	105	SRR22784529	5,209,535	2	5,015,328	50.5	Unicycler	Hague
9503_1L1	B2	EC_9503_1L1_D	GCA_027274295.1	48,370,398	150	1,393	SRR22784693	13,658	8,550	22	SRR22784692	5,209,556	2	5,015,349	50.5	Unicycler	Hague
9503_1S2	B2	EC_9503_1S2_D	GCA_027278645.1	55,364,286	150	1,594	SRR22784775	22,746	6,681	29	SRR22784774	5,209,556	2	5,015,349	50.5	Unicycler	Hague
9503_2H1	B2	EC_9503_2H1_D	GCA_027278755.1	36,904,020	150	966	SRR22784952	16,386	14,043	40	SRR22784951	5,729,051	7	5,064,243	50.5	Unicycler	Hague
9503_C1	B2	EC_9503_C1_H	GCA_027276465.1	21,775,344	150	609	SRR22784667	198,070	10,657	394	SRR22784666	5,362,005	79	1,389,246	50.5	SPAdes	Hague
9503_C2	B2	EC_9503_C2_H	GCA_027274565.1	21,479,264	150	629	SRR22784460	857	5,675	1	SRR22784459	5,123,787	60	245,302	50.5	Unicycler	Hague
9503_C3	B1	EC_9503_C3_H	GCA_027276765.1	30,303,720	150	880	SRR22784725	72,677	10,654	150	SRR22784724	5,167,524	22	3,702,254	50.5	SPAdes	Hague
9619_1H1	B1	EC_9619_1H1_D	GCA_027274625.1	17,160,058	150	463	SRR22784457	126,821	6,337	145	SRR22784456	5,554,269	2	5,435,017	50.5	Unicycler	Grandora-3
9619_1H3	D	EC_9619_1H3_D	GCA_027274345.1	32,346,874	150	921	SRR22784803	51,282	12,872	125	SRR22784801	5,265,929	5	5,192,593	50.5	Unicycler	Grandora-3
9619_1S3	B1	EC_9619_1S3_D	GCA_027278745.1	53,107,576	150	1,512	SRR22784773	313	8,346	0	SRR22784772	5,268,276	132	135,745	50.5	Unicycler	Grandora-3
9619_2H1	U	EC_9619_2H1_D	GCA_027276925.1	21,108,892	150	603	SRR22784742	117,134	7,991	178	SRR22784741	5,250,611	3	3,321,052	50.8	Flye_Medaka_Pilon	Grandora-3
9619_2L1	G	EC_9619_2L1_D	GCA_027279515.1	26,460,574	150	733	SRR22784780	116,930	5,559	120	SRR22784769	5,411,232	3	5,211,599	50.8	Unicycler	Grandora-3
9619_2S1	B1	EC_9619_2S1_D	GCA_027276865.1	39,639,240	150	1,070	SRR22784848	94,111	6,159	104	SRR22784847	5,550,411	11	2,830,275	50.6	Unicycler	Grandora-3
9619_3L1	A	EC_9619_3L1_D	GCA_027273835.1	41,232,434	150	1,254	SRR22784708	309	9,096	1	SRR22784707	4,927,596	160	94,428	50.5	Unicycler	Grandora-3
9619_3S1	A	EC_9619_3S1_D	GCA_027276205.1	26,959,044	150	761	SRR22784907	148,340	5,752	161	SRR22784906	5,310,753	4	4,932,041	50.4	Unicycler	Grandora-3
9619_C1	A	EC_9619_C1_H	GCA_027271535.1	28,036,666	150	782	SRR22784781	153,664	6,095	174	SRR22784501	5,373,513	4	5,205,352	50.4	Unicycler	Grandora-3
9619_C6	B1	EC_9619_C6_H	GCA_027278865.1	28,873,048	150	828	SRR22784763	134,151	6,014	154	SRR22784762	5,226,245	4	5,107,832	50.6	Unicycler	Grandora-3
9619_C8	A	EC_9619_C8_H	GCA_027278345.1	26,957,664	150	743	SRR22784914	68,470	6,081	77	SRR22784913	5,433,915	20	3,754,749	50.6	Unicycler	Grandora-3
E1F_1	D	EC_E1F_1_E	GCA_027275705.1	34,723,672	150	952	SRR22784852	33,596	8,021	49	SRR22784851	5,473,166	19	1,172,182	50.4	Unicycler	Hepburn-2
E1F_3	D	EC_E1F_3_E	GCA_027279395.1	48,415,780	150	1,355	SRR22784561	60,299	5,911	67	SRR22784550	5,357,844	3	5,291,311	50.5	Flye_Medaka_Pilon	Hepburn-2
E1L_1	D	EC_E1L_1_E	GCA_027276295.1	9,296,650	150	260	SRR22784886	19,228	8,725	31	SRR22784885	5,367,646	16	2,996,644	50.5	Unicycler	Hepburn-2
E1L_3	D	EC_E1L_3_E	GCA_027273995.1	56,529,616	150	1,585	SRR22784578	26,153	6,150	30	SRR22784577	5,347,294	27	2,945,799	50.4	SPAdes	Hepburn-2
E2DI_2	F	EC_E2DI_2_E	GCA_027274655.1	16,993,326	150	410	SRR22784449	24,990	10,686	43	SRR22784448	6,215,850	24	5,759,448	50.3	Unicycler	Grandora
E2DN_1	B1	EC_E2DN_1_E	GCA_027271415.1	17,081,166	150	502	SRR22784669	32,718	9,452	61	SRR22784668	5,104,713	6	4,877,570	50.6	Flye_Medaka_Pilon	Grandora
E2L_1	A	EC_E2L_1_E	GCA_027279165.1	11,094,896	150	336	SRR22784574	52,766	9,898	105	SRR22784841	4,957,218	3	4,786,389	50.7	Flye_Medaka_Pilon	Grandora
E2P_1	A	EC_E2P_1_E	GCA_027276835.1	11,438,750	150	280	SRR22784861	17,317	8,211	23	SRR22784860	6,134,691	115	152,646	50.5	Unicycler	Grandora
E2BE_2	A	EC_E2BE_2_E	GCA_027275085.1	13,876,038	150	398	SRR22784519	6,857	9,354	12	SRR22784518	5,227,862	22	627,135	50.8	Unicycler	Grandora
E3BE_1	D	EC_E3BE_1_E	GCA_027275885.1	18,485,222	150	529	SRR22784876	6,356	8,759	11	SRR22784875	5,244,186	18	1,217,060	50.4	Unicycler	Vanscoy
E3DI_1	A	EC_E3DI_1_E	GCA_027279465.1	29,605,364	150	895	SRR22784857	14,145	9,530	27	SRR22784846	4,961,055	25	3,452,736	50.7	SPAdes	Vanscoy
E3F_1	U	EC_E3F_1_E	GCA_027275555.1	12,375,448	150	359	SRR22784607	20,972	8,200	33	SRR22784606	5,169,908	73	268,625	51.0	Unicycler	Vanscoy
E4BE_1	A	EC_E4BE_1_E	GCA_027276785.1	13,461,708	150	366	SRR22784629	12,915	10,810	25	SRR22784628	5,518,184	4	5,361,954	50.5	Flye_Medaka_Pilon	Saskatoon-3
E4BF_2	A	EC_E4BF_2_E	GCA_027276125.1	30,695,136	150	906	SRR22784877	37,492	4,533	33	SRR22784662	5,082,846	3	4,928,583	50.6	Unicycler	Saskatoon-3
E4DI_1	Cryptic	EC_E4DI_1_E	GCA_027278625.1	20,827,002	150	583	SRR22784964	36,029	7,674	52	SRR22784963	5,362,134	3	5,220,686	50.4	Flye_Medaka_Pilon	Saskatoon-3
E4F_1	A	EC_E4F_1_E	GCA_027278275.1	22,069,950	150	619	SRR22784939	16,040	7,366	22	SRR22784938	5,350,879	12	2,678,522	50.5	Flye_Medaka_Pilon	Saskatoon-3
E4F_2	A	EC_E4F_2_E	GCA_027278265.1	15,338,944	150	424	SRR22784757	9,738	10,786	19	SRR22784756	5,429,528	21	5,183,274	50.5	Unicycler	Saskatoon-3
E4FP_1	A	EC_E4FP_1_E	GCA_027278895.1	7,386,514	150	200	SRR22784483	7,508	9,385	13	SRR22784482	5,543,084	52	4,231,916	50.7	SPAdes	Saskatoon-3
E4L_1	A	EC_E4L_1_E	GCA_027273905.1	33,442,382	150	902	SRR22784824	19,585	7,321	26	SRR22784823	5,560,870	30	2,939,255	50.5	SPAdes	Saskatoon-3
E4P_1	A	EC_E4P_1_E	GCA_027278565.1	40,899,410	150	1,302	SRR22784947	17,645	8,755	33	SRR22784946	4,712,909	4	4,564,743	50.7	Flye_Medaka_Pilon	Saskatoon-3
E5BE_1	C	EC_E5BE_1_E	GCA_027274695.1	19,752,646	150	609	SRR22784453	78,779	8,605	139	SRR22784452	4,866,911	3	4,767,191	50.6	Flye_Medaka_Pilon	Wakaw-2
E5BF_1	C	EC_E5BF_1_E	GCA_027278435.1	20,218,240	150	581	SRR22784929	71,334	8,037	110	SRR22784928	5,217,075	4	5,077,956	50.6	Flye_Medaka_Pilon	Wakaw-2
E5BF_2	A	EC_E5BF_2_E	GCA_027274455.1	12,587,196	150	345	SRR22784589	56,353	10,152	105	SRR22784588	5,471,483	14	3,948,144	50.5	Unicycler	Wakaw-2
E5DN_1	B1	EC_E5DN_1_E	GCA_027276605.1	24,302,406	150	628	SRR22784719	14,181	5,572	14	SRR22784718	5,804,111	77	375,403	50.7	Unicycler	Wakaw-2
E5F_2	A	EC_E5F_2_E	GCA_027274895.1	24,863,154	150	722	SRR22784547	11,125	10,587	23	SRR22784546	5,166,466	5	5,034,824	50.5	Unicycler	Wakaw-2
E5L_1	A	EC_E5L_1_E	GCA_027274415.1	37,609,590	150	1,082	SRR22784503	78,953	9,049	137	SRR22784694	5,209,459	35	3,120,925	50.4	SPAdes	Wakaw-2
E6BE_1	A	EC_E6BE_1_E	GCA_027275685.1	12,777,396	150	393	SRR22784870	12,322	6,005	15	SRR22784869	4,881,313	6	4,784,173	50.4	Flye_Medaka_Pilon	Hepburn
E6DI_1	D	EC_E6DI_1_E	GCA_027276105.1	12,275,620	150	307	SRR22784661	16,804	4,555	13	SRR22784660	6,005,238	29	3,073,705	50.4	Unicycler	Hepburn
E6DN_1	A	EC_E6DN_1_E	GCA_027276035.1	36,531,016	150	1,132	SRR22784651	58,144	5,842	70	SRR22784650	4,839,665	7	4,758,794	50.6	Flye_Medaka_Pilon	Hepburn
E6FP_1	D	EC_E6FP_1_E	GCA_027279345.1	18,422,972	150	464	SRR22784903	36,086	5,425	33	SRR22784891	5,957,830	25	5,198,894	50.4	Unicycler	Hepburn
E7BE_2	A	EC_E7BE_2_E	GCA_027275805.1	66,031,578	150	1,987	SRR22784641	34,972	3,709	26	SRR22784640	4,985,745	4	4,773,874	50.7	Flye_Medaka_Pilon	Delisle
E7DI_1	D	EC_E7DI_1_E	GCA_027275195.1	29,997,864	150	735	SRR22784689	72,468	4,390	52	SRR22784688	6,122,945	21	3,565,206	50.2	Unicycler	Delisle
E7DN_1	D	EC_E7DN_1_E	GCA_027274525.1	21,826,826	150	646	SRR22784444	38,688	8,887	68	SRR22784443	5,067,326	2	5,019,757	50.5	Flye_Medaka_Pilon	Delisle
E7FP_1	E	EC_E7FP_1_E	GCA_027274555.1	12,684,924	150	348	SRR22784595	88,228	3,713	60	SRR22784594	5,464,077	8	4,257,333	50.7	Flye_Medaka_Pilon	Delisle
E7L_1	B1	EC_E7L_1_E	GCA_027273825.1	14,193,372	150	395	SRR22784440	7,385	4,825	7	SRR22784439	5,388,079	129	124,700	50.5	Unicycler	Delisle
E7P_1	D	EC_E7P_1_E	GCA_027274825.1	25,200,408	150	616	SRR22784556	35,875	4,867	28	SRR22784555	6,134,689	17	3,154,018	50.4	Unicycler	Delisle
E8BE_1	D	EC_E8BE_1_E	GCA_027271405.1	24,041,092	150	681	SRR22784809	14,221	9,673	26	SRR22784808	5,293,832	6	5,167,750	50.4	Unicycler	Hague-2
E8BF_1	A	EC_E8BF_1_E	GCA_027273965.1	33,993,240	150	847	SRR22784833	18,921	10,240	32	SRR22784832	6,019,164	32	5,502,525	50.8	Unicycler	Hague-2
E8BF_2	A	EC_E8BF_2_E	GCA_027276645.1	50,832,324	150	1,257	SRR22784865	47,197	4,180	33	SRR22784864	6,067,449	17	3,598,239	50.8	Flye_Medaka_Pilon	Hague-2
E8FP_1	D	EC_E8FP_1_E	GCA_027278635.1	17,150,798	150	489	SRR22784950	21,298	10,908	44	SRR22784949	5,258,086	4	5,192,010	50.5	Unicycler	Hague-2
E8FP_2	D	EC_E8FP_2_E	GCA_027274845.1	41,355,804	150	1,178	SRR22784560	73,513	8,821	123	SRR22784559	5,267,695	4	5,201,619	50.5	Unicycler	Hague-2
E8L_1	D	EC_E8L_1_E	GCA_027275065.1	16,697,808	150	474	SRR22784425	34,222	10,499	68	SRR22784424	5,282,459	4	5,157,959	50.4	Unicycler	Hague-2
E9BE_1	A	EC_E9BE_1_E	GCA_027275025.1	22,841,958	150	640	SRR22784517	95,631	7,019	125	SRR22784515	5,351,441	14	5,215,244	50.5	Unicycler	Saskatoon-4
E9DI_1	A	EC_E9DI_1_E	GCA_027279245.1	21,843,278	150	598	SRR22784421	15,933	13,985	41	SRR22784410	5,473,347	25	5,306,572	50.7	SPAdes	Saskatoon-4
E9DI_2	A	EC_E9DI_2_E	GCA_027276475.1	54,356,334	150	1,513	SRR22784699	32,634	8,653	52	SRR22784698	5,390,511	2	5,292,972	50.7	Flye_Medaka_Pilon	Saskatoon-4
E9DN_1	A	EC_E9DN_1_E	GCA_027276225.1	22,534,206	150	616	SRR22784882	26,780	12,285	60	SRR22784881	5,488,322	4	5,473,902	50.5	Unicycler	Saskatoon-4
E9DN_2	A	EC_E9DN_2_E	GCA_027274035.1	19,168,758	150	511	SRR22784839	20,376	9,773	35	SRR22784838	5,631,978	7	4,603,841	50.5	Unicycler	Saskatoon-4
E9FP_1	A	EC_E9FP_1_E	GCA_027275945.1	32,155,320	150	1,006	SRR22784632	22,233	12,280	57	SRR22784631	4,792,886	14	4,234,197	50.7	SPAdes	Saskatoon-4
E9L_1	D	EC_E9L_1_E	GCA_027275395.1	21,414,904	150	617	SRR22784411	18,872	12,402	45	SRR22784409	5,209,151	6	5,193,018	50.5	Unicycler	Saskatoon-4
E10BF_1	C	EC_E10BF_1_E	GCA_027273915.1	20,826,990	150	618	SRR22784826	19,189	6,740	26	SRR22784825	5,051,410	13	4,296,521	50.8	Flye_Medaka_Pilon	Hague
E10BF_2	C	EC_E10BF_2_E	GCA_027278115.1	23,318,572	150	688	SRR22784752	69,655	4,358	60	SRR22784751	5,084,811	11	2,966,714	50.7	Flye_Medaka_Pilon	Hague
E10FP_1	A	EC_E10FP_1_E	GCA_027275525.1	23,257,250	150	701	SRR22784605	94,237	9,162	173	SRR22784604	4,979,656	3	4,770,377	50.7	Flye_Medaka_Pilon	Hague
E10L_1	E	EC_E10L_1_E	GCA_027276625.1	21,148,566	150	547	SRR22784721	40,743	8,024	56	SRR22784720	5,803,523	58	382,382	50.2	SPAdes	Hague
E10L_2	D	EC_E10L_2_E	GCA_027274225.1	20,102,204	150	603	SRR22784676	31,132	9,811	61	SRR22784675	5,002,823	12	4,909,646	50.6	SPAdes	Hague
E11BE_1	D	EC_E11BE_1_E	GCA_027278395.1	20,196,834	150	571	SRR22784917	24,083	7,012	32	SRR22784916	5,309,564	4	5,247,800	50.5	Unicycler	Dalmeny-2
E11BF_1	A	EC_E11BF_1_E	GCA_027276445.1	9,797,308	150	274	SRR22784701	36,956	8,341	58	SRR22784700	5,354,887	61	481,722	50.7	SPAdes	Dalmeny-2
E11DN_1	C	EC_E11DN_1_E	GCA_027275185.1	23,842,388	150	715	SRR22784714	38,955	8,900	69	SRR22784713	4,998,858	5	3,083,879	50.8	Flye_Medaka_Pilon	Dalmeny-2
E11FP_1	Cryptic	EC_E11FP_1_E	GCA_027275165.1	16,213,528	150	501	SRR22784710	14,715	9,345	28	SRR22784709	4,850,940	4	1,656,476	50.9	Flye_Medaka_Pilon	Dalmeny-2
E11L_1	B1	EC_E11L_1_E	GCA_027276485.1	12,496,600	150	374	SRR22784466	40,562	7,819	63	SRR22784465	5,006,213	3	4,943,600	50.6	Flye_Medaka_Pilon	Dalmeny-2
E12BE_1	B1	EC_E12BE_1_E	GCA_027279185.1	25,621,364	150	737	SRR22784502	98,971	9,230	175	SRR22784495	5,213,742	4	4,792,197	50.6	Unicycler	Saskatoon-5
E12BF_2	G	EC_E12BF_2_E	GCA_027275585.1	26,790,620	150	653	SRR22784867	68,008	9,937	110	SRR22784866	6,152,158	131	124,472	50.7	Unicycler	Saskatoon-5
E12DN_1	D	EC_E12DN_1_E	GCA_027273585.1	14,275,372	150	417	SRR22784538	114,942	8,628	193	SRR22784537	5,139,694	3	5,063,323	50.7	Unicycler	Saskatoon-5
E12F_1	D	EC_E12F_1_E	GCA_027274045.1	17,055,298	150	498	SRR22784842	139,115	9,148	248	SRR22784840	5,139,547	3	5,063,167	50.7	Unicycler	Saskatoon-5
E12F_2	A	EC_E12F_2_E	GCA_027273535.1	11,154,108	150	340	SRR22784413	48,218	9,020	88	SRR22784412	4,918,815	2	4,818,391	50.7	Unicycler	Saskatoon-5
E13BF_1	A	EC_E13BF_1_E	GCA_027278105.1	18,211,186	150	455	SRR22784921	78,986	7,694	101	SRR22784920	6,004,959	7	5,594,201	50.6	Flye_Medaka_Pilon	Biggar-2
E13BF_2	A	EC_E13BF_2_E	GCA_027273645.1	42,010,674	150	1,226	SRR22784817	27,652	6,268	34	SRR22784816	5,140,365	6	4,818,705	50.6	Unicycler	Biggar-2
E13BF_4	B1	EC_E13BF_4_E	GCA_027276405.1	18,170,370	150	521	SRR22784571	5,310	5,343	5	SRR22784570	5,227,896	26	535,968	50.6	Unicycler	Biggar-2
E13DN_1	A	EC_E13DN_1_E	GCA_027278805.1	36,248,520	150	1,080	SRR22784771	88,909	7,494	132	SRR22784770	5,032,311	3	4,837,947	50.5	Unicycler	Biggar-2
E13DN_4	A	EC_E13DN_4_E	GCA_027274325.1	30,567,288	150	911	SRR22784678	123,335	6,547	160	SRR22784677	5,032,310	3	4,837,946	50.5	Unicycler	Biggar-2
E13DN_5	A	EC_E13DN_5_E	GCA_027275825.1	21,995,262	150	656	SRR22784874	33,333	6,529	43	SRR22784873	5,030,179	14	3,301,583	50.5	SPAdes	Biggar-2
E13FP_1	A	EC_E13FP_1_E	GCA_027276905.1	18,445,678	150	508	SRR22784601	56,432	8,410	87	SRR22784600	5,447,659	6	5,246,920	50.5	Unicycler	Biggar-2
E13FP_2	D	EC_E13FP_2_E	GCA_027278705.1	28,530,658	150	844	SRR22784954	126,550	7,327	183	SRR22784953	5,068,664	20	1,750,506	50.5	SPAdes	Biggar-2
E13FP_3	A	EC_E13FP_3_E	GCA_027276345.1	36,781,762	150	1,117	SRR22784837	87,896	7,719	137	SRR22784836	4,938,820	24	4,770,194	50.5	SPAdes	Biggar-2
E13L_1	B1	EC_E13L_1_E	GCA_027278965.1	14,556,682	150	419	SRR22784777	92,633	8,619	153	SRR22784776	5,207,291	3	5,001,890	50.6	Unicycler	Biggar-2
E13L_2	B1	EC_E13L_2_E	GCA_027274735.1	34,861,132	150	982	SRR22784462	95,306	6,690	120	SRR22784461	5,323,037	30	4,960,876	50.6	SPAdes	Biggar-2
E13L_3	A	EC_E13L_3_E	GCA_027276065.1	30,821,026	150	894	SRR22784894	21,512	4,623	19	SRR22784893	5,169,821	34	4,806,028	50.6	SPAdes	Biggar-2
E14BF_1	A	EC_E14BF_1_E	GCA_027274805.1	24,284,436	150	693	SRR22784545	79,294	8,116	122	SRR22784544	5,255,581	4	4,895,773	50.4	Flye_Medaka_Pilon	Herbert
E14F_4	A	EC_E14F_4_E	GCA_027274965.1	41,209,512	150	1,142	SRR22784835	36,437	7,392	50	SRR22784834	5,409,716	29	5,263,650	50.4	SPAdes	Herbert
E15BF_1	G	EC_E15BF_1_E	GCA_027275925.1	30,075,172	150	834	SRR22784896	145,025	7,622	204	SRR22784895	5,404,178	25	1,916,113	50.8	SPAdes	Delisle-2
E15BF_3	G	EC_E15BF_3_E	GCA_027273745.1	34,358,658	150	917	SRR22784796	12,883	5,442	12	SRR22784795	5,615,681	118	265,165	50.7	SPAdes	Delisle-2
E15DN_1	Cryptic	EC_E15DN_1_E	GCA_027275985.1	26,056,554	150	705	SRR22784647	15,509	5,159	14	SRR22784646	5,545,560	41	993,052	50.5	Flye_Medaka_Pilon	Delisle-2
E15F_2	G	EC_E15F_2_E	GCA_027279215.1	32,742,580	150	881	SRR22784829	9,951	7,597	14	SRR22784818	5,567,882	18	4,680,733	50.8	SPAdes	Delisle-2
E15F_3	G	EC_E15F_3_E	GCA_027278145.1	23,442,592	150	650	SRR22784736	12,762	7,298	17	SRR22784735	5,410,285	7	5,213,851	50.8	Unicycler	Delisle-2
E15FP_2	D	EC_E15FP_2_E	GCA_027276745.1	23,778,464	150	603	SRR22784657	33,174	8,933	50	SRR22784656	5,912,141	80	479,906	50.3	SPAdes	Delisle-2
E15L_1	B1	EC_E15L_1_E	GCA_027279565.1	20,035,172	150	590	SRR22784492	15,289	6,889	21	SRR22784481	5,085,641	30	3,324,430	50.7	SPAdes	Delisle-2
E15L_6	A	EC_E15L_6_E	GCA_027278885.1	36,660,412	150	1,079	SRR22784759	21,828	6,336	27	SRR22784969	5,094,836	50	3,083,087	50.5	SPAdes	Delisle-2
E16BE_1	G	EC_E16BE_1_E	GCA_027273785.1	25,348,740	150	660	SRR22784552	11,381	8,661	17	SRR22784551	5,758,716	13	2,245,761	50.7	Unicycler	Aberdeen
E16BF_1	G	EC_E16BF_1_E	GCA_027274165.1	19,805,636	150	549	SRR22784671	12,581	8,339	19	SRR22784670	5,411,710	3	5,213,640	50.8	Unicycler	Aberdeen
E16DI_1	A	EC_E16DI_1_E	GCA_027279575.1	23,430,138	150	640	SRR22784830	50,544	7,712	71	SRR22784505	5,494,747	61	967,358	50.5	SPAdes	Aberdeen
E16DI_3	Cryptic	EC_E16DI_3_E	GCA_027278605.1	34,747,070	150	919	SRR22784943	42,604	7,884	59	SRR22784942	5,670,382	16	5,463,737	50.1	SPAdes	Aberdeen
E16F_1	G	EC_E16F_1_E	GCA_027274195.1	30,834,832	150	855	SRR22784509	98,574	8,669	158	SRR22784508	5,404,034	27	3,915,774	50.8	SPAdes	Aberdeen
E16F_5	G	EC_E16F_5_E	GCA_027271435.1	30,365,990	150	818	SRR22784807	216,942	7,543	294	SRR22784806	5,566,312	20	4,303,474	50.8	SPAdes	Aberdeen
E16P_1	G	EC_E16P_1_E	GCA_027275785.1	31,111,712	150	758	SRR22784859	11,138	8,497	15	SRR22784858	6,160,380	46	611,900	50.3	Flye_Medaka_Pilon	Aberdeen

a The phylogroup was determined by analyzing whole-genome assemblies from each isolate using the EzClermont program; isolates classified as “cryptic” (*n* = 6) did not fit any phylogroup designation.

b The GenBank name denotes several parameters for each isolate: EC was used to designate E. coli; the last letter of the name string denotes the source (D, disease; H, healthy; E, environmental). For disease and healthy isolates, the 4- or 5-digit number represents a different colibacillosis outbreak, where isolates from dead birds heavily colonized by E. coli and healthy birds were obtained from the same flocks; the letter represents the source organ (H, heart; L, liver; S, spleen; B, bone marrow; C, cecum); the preceding number represents the bird number; the following number was used for isolate identification. Environmental E. coli strains were isolated from 8 different sources within Saskatchewan poultry barns (F, feed; FP, feed pan; L, litter; BF, barn floor; BE, barn entrance; DI, drinker interior; DN, drinker nipple; P, pests); the preceding number represents the farm number, and the following number was used for isolate identification.

c Name of the town within the province of Saskatchewan where each farm is located; the numerical designation signifies a different broiler farm within the same area.

Samples were homogenized (MM400; Retsch) for 5 min at 30 Hz in tubes containing 1 mL phosphate-buffered saline (PBS) and a 5-mm steel bead. Aliquots were grown overnight on MacConkey agar at 37°C; suspected E. coli colonies (*n* = 895) were confirmed via positive indole and negative Simmons-citrate biochemical tests. Isolates chosen based on their antibiotic resistance patterns and biofilm phenotype (7) were grown overnight at 37°C in Luria broth. Genomic DNA (gDNA) was purified (GenElute bacterial genomic DNA kit; NA2120; Sigma-Aldrich) and sequenced on the Oxford Nanopore MinION (in house) and Illumina NovoSeq (Novogene, Sacramento, CA) platforms. For Nanopore, the addition of 0.4× (vol/vol) magnetic beads (MN-744970.50; Macherey-Nagel) was used to purify fragments of >500 bp; detailed sequencing protocols are described elsewhere (7). For Illumina, gDNA was enzymatically fragmented following the Ultra II FS DNA library prep kit protocol (E7805L; New England Biolabs); >500-bp fragments were removed (as above), and 400- to 500-bp fragments were purified by the addition of 0.2× magnetic beads. The samples were assessed for quality and purity (DS-11 FX spectrophotometer/fluorometer; FroggaBio) and proper size range (Bioanalyzer with high-sensitivity DNA chip; 5067-4626; Agilent Technologies).

The read number and quality were assessed for the Nanopore (NanoStat v1.5.0) (8) and Illumina (FastQC v0.11.9) (9) reads. The Nanopore reads were base called using MinKNOW v2.0 (10) and Guppy v2.1.1 (11). The reads were trimmed and filtered using Porechop v0.2.4 (12) and Filtlong v0.2.1 (13) for Nanopore and fastp v0.20.1 (14) for Illumina. Long-read assemblies produced using Flye v2.9 (15) were used for hybrid assembly using Unicycler v0.5.0 (*n* = 156) (16) or SPAdes v3.15.4 (*n* = 49) (17). The hybrid assemblies were polished with the short reads using Pilon v1.24 (18) (with MUMmer v4.0.0 [19] and SAMtools v1.13 [20]) and Snippy v4.6.0 (21) (with VCFtools v0.1.16 [22] and Tabix v0.2.6 [20]). The assembly quality was assessed using QUAST v5.0.2 (23). For isolates that did not generate a hybrid assembly (*n* = 40), the Flye assemblies were polished using Medaka v0.12.1 (24) and Pilon, and the read contamination was assessed using ConFindr v0.7.4 (25). Default parameters were used for all software.

Genomes were annotated using the NCBI Prokaryotic Genome Annotation Pipeline v6.4 (26). The average length of the E. coli genomes was 5,318,463 bp, compiled from an average of 66,595 long reads (coverage, 92×) and 28,666,849 short reads (coverage, 811×) (Table 1). The average *N*_50_ value of the genomes was 3,786,935 bp, with an average of 22.3 contigs. Some genomes are likely to be complete, but we did not determine their circularity. E. coli phylogroups were identified using EzClermont v0.7.0 (27); the most abundant group was A (*n* = 97). Analysis of this collection should reveal potential relationships between disease-causing, commensal, and environmental E. coli isolates.

### Data availability.

All of the hyperlinked accession numbers for the raw reads and assemblies are summarized in Table 1 and available under BioProject accession number PRJNA912639.
